# The Role of Advanced Glycation End-Products in the Pathophysiology and Pharmacotherapy of Cardiovascular Disease

**DOI:** 10.3390/ijms26157311

**Published:** 2025-07-29

**Authors:** Karina O. Mota, Carla M. L. de Vasconcelos, Lorrie A. Kirshenbaum, Naranjan S. Dhalla

**Affiliations:** 1 Department of Physiology, Center of Biological and Health Sciences, Federal University of Sergipe, São Cristóvão 49100-000, Brazil; koliveiramota@sbrc.ca (K.O.M.); carlamlv@academico.ufs.br (C.M.L.d.V.); 2 Institute of Cardiovascular Sciences, St. Boniface Hospital Albrechtsen Research Centre, Department of Physiology and Pathophysiology, Max Rady College of Medicine, University of Manitoba, Winnipeg, MB R2H 2A6, Canada; lkirshenbaum@sbrc.ca

**Keywords:** advanced glycation end-products (AGEs), AGE receptors (RAGEs), soluble RAGEs, AGE–RAGE axis, cardiovascular diseases

## Abstract

Advanced glycation end-products (AGEs) are formed by the non-enzymatic glycation of proteins, lipids, and nucleic acids due to the consumption of high-carbohydrate diets; their production is also promoted by a sedentary lifestyle as well as cigarette smoking. Elevated levels of AGEs in the circulatory system and internal organs of the body are commonly observed in a number of cardiovascular diseases such as hypertension, diabetes, atherosclerosis, coronary artery disease, aortic aneurysm, atrial fibrillation, myocardial infarction, and heart failure, which are associated with the development of oxidative stress and myocardial inflammation. The adverse effects of AGEs on the cardiovascular system are elicited by both non-receptor mechanisms involving the cross-linking of extracellular and intracellular proteins, and by receptor-mediated mechanisms involving the binding of AGEs with advanced glycation end-product receptors (RAGEs) on the cell membrane. AGE–RAGE interactions along with the cross-linking of proteins promote the generation of oxidative stress, the production of inflammation, the occurrence of intracellular Ca^2+^-overload, and alterations in the extracellular matrix leading to the development of cardiovascular dysfunction. AGEs also bind with two other protein receptors in the circulatory system: soluble RAGEs (sRAGEs) are released upon the proteolysis of RAGEs due to the activation of matrix metalloproteinase, and endogenous secretory RAGEs (esRAGEs) are secreted as a spliced variant of endogenous RAGEs. While the AGE–RAGE signal transduction axis serves as a pathogenic mechanism, both sRAGEs and esRAGEs serve as cytoprotective interventions. The serum levels of sRAGEs are decreased in ischemic heart disease, vascular disease, and heart failure, as well as in other cardiovascular diseases, but are increased in chronic diabetes and renal disease. Several interventions which can reduce the formation of AGEs, block the AGE–RAGE axis, or increase the levels of circulating sRAGEs have been shown to exert beneficial effects in diverse cardiovascular diseases. These observations support the view that the AGE–RAGE axis not only plays a critical role in pathogenesis, but is also an excellent target for the treatment of cardiovascular disease.

## 1. Introduction

Cardiovascular disease is a major cause of morbidity and mortality worldwide, and its prevalence is commonly associated with lifestyle and nutrition [1,2,3,4,5]. Since diet and lifestyle are the main exogenous sources of advanced glycation end-products (AGEs), elevated levels of circulating AGEs as well as their accumulation in the heart and blood vessels are linked to the progression of cardiovascular complications in ischemic heart disease, atherosclerosis, hypertension, diabetes, and heart failure. In particular, high levels of AGEs are produced upon the consumption of excessive amounts of high-carbohydrate or hypercaloric diets and high-temperature-cooked foods, as well as by cigarette smoking and a sedentary lifestyle [6,7,8,9]. On the other hand, endogenous AGEs are produced and accumulate slowly during aging and in several pathological conditions. Such diseases are associated with oxidative stress, inflammation, and hyperglycemia, and are known to lead to the development of cardiovascular dysfunction [7,10,11]. Although more than 20 AGEs are known to be present in the human body, the classification, composition, and protein targets of just some of these important AGEs are summarized in Table 1.

In general, AGEs are a group of diverse and chemically heterogeneous compounds which are formed from different precursors in two pathways—the Maillard reaction and the Polyol pathway [9]. The Maillard pathway is the principal source of AGEs; this comprises the condensation of a carbonyl group from a reducing sugar and an amine group from moieties such as the side chains of lysine or arginine [12]. This reaction produces a Schiff base, which undergoes Amadori rearrangement, resulting in stable products [6,13]. These metabolites are considered to be early glycation products, such as glucose-derived AGEs, which act as precursors for AGEs (created during the Maillard reaction), along with a heterogeneous class of reactive carbonyl intermediates such as glyoxal, methylglyoxal, glyceraldehyde, glycolaldehyde, diacetyl, and 1 and 3-deoxyglucosone [14,15]. On the other hand, the Polyol pathway produces AGEs upon the reduction of glucose to sorbitol by aldose reductase using nicotinamide adenine dinucleotide phosphate (NADPH) as a cofactor. The sorbitol is then reduced to fructose by sorbitol dehydrogenase, and this reaction results in the formation of α-dicarbonyl compounds and AGEs in the heart [16]. It should be pointed out that increased oxidative stress leads to the oxidation of monosaccharides in the presence of phosphates and transition metal ions during a process known as direct autoxidation reaction, which results in the formation of α-dicarbonyl compounds [17]. Although reactive carbonyls are generated under physiological conditions, these compounds are maintained at a minimal level due to degradation. However, when their elimination pathway is compromised, levels of these compounds rise, leading to the accumulation of AGEs.

Several AGEs resulting from alterations in the incorporation of lysine and/or arginine amino groups, including carboxymethyl lysine, carboxyethyl lysine, and argpyrimidine, have also been identified in several diseases [18,19]. It should also be noted that methylglyoxal modifies heat shock protein 27 (HSP27), leading to the formation of argpyrimidine; moreover, the modification of HSP27 is increased in diabetic failing hearts as a result [20]. The accumulation of AGEs in several organs of the body contributes to the development and progression of various diseases, including cardiac, renal, and peripheral vascular ailments [21,22]. The pathological impacts of AGEs stem from their capacity to induce oxidative stress and inflammation by interactions with cell-surface-bound AGE receptors (RAGEs) or the formation of cross-links with cellular proteins, thereby modifying cellular structure and function [23]. By triggering oxidative stress, AGEs instigate the activation of various stress-induced transcription factors, resulting in the synthesis of pro-inflammatory and inflammatory mediators like cytokines and acute-phase proteins.

Several mechanisms have been proposed to explain the adverse effects of AGEs in diseased myocardium; these include cross-linking with tissue extracellular and intracellular proteins, as well as binding to RAGEs [24]. It is noteworthy that the cross-linking of AGEs alters the extracellular matrix proteins through intramolecular covalent bonds leading to a decrease in vascular elasticity, myocardial flexibility, and promotion of vascular and myocardial stiffness [25]. Furthermore, the cross-linking of AGEs with intracellular sarcoplasmic reticulum proteins, such as Ca^2+^-pump (SERCA) and Ca^2+^-release channels or ryanodine receptor (RyR), leads to alterations in Ca^2+^ homeostasis and the depression of cardiac contractility due to hemodynamic overload or myocardial injury [26,27]. It should also be noted that RAGEs are members of the immunoglobulin superfamily and are expressed in cardiomyocytes, vascular smooth muscle, macrophages, lymphocytes, and endothelial cells. AGE–RAGE interaction can modulate inflammatory responses by increasing the expression of NF-κB, oxyradical formation, and the production of pro-inflammatory cytokines, as well as growth factors [28]. Thus, it has been claimed that both receptor-mediated and non-receptor-mediated mechanisms involving AGE–RAGE interaction and cross-linking with proteins are involved in the induction of cardiac dysfunction during the development of various cardiovascular diseases [29,30,31,32,33,34].

Elevated levels of AGEs in both serum and myocardium were found to be involved in a wide variety of cardiovascular diseases such as hypertension, diabetes, atherosclerosis, myocardial infarction, coronary artery disease, heart failure, atrial fibrillation, and aortic aneurysm [29,30,31,32,33,34,35,36,37,38,39,40]. It was also found that interventions which exert beneficial effects in various cardiovascular disorders also lower the serum and myocardial levels of AGEs. This study was undertaken in order to provide an updated comprehensive review of the involvement of AGEs in the pathogenesis and pharmacotherapy of cardiovascular dysfunction in various cardiovascular diseases. In particular, the role of AGEs in the development of ischemic heart disease, vascular abnormalities, heart failure, and diabetic cardiomyopathy will be discussed. This article also plans to provide information on the sources and formation of AGEs, as well as the presence of endogenous AGE antagonists in the circulatory system. Furthermore, the function of AGE–RAGE interactions and AGE–RAGE signal transduction mechanisms in cardiovascular diseases will be described. It should be mentioned that appropriate literature for this article was found by searching on MEDLINE via PubMed, using search terms such as AGEs and cardiovascular disease, RAGEs and cardiovascular disease, AGEs and signal transduction, AGEs and oxidative stress, AGEs and inflammation, AGEs and Ca^2+^-overload, and AGEs and cross-linked proteins. The articles cited in this review were selected in order to provide information on the involvement of the AGE–RAGE axis, as well as AGE and cross-linked protein interactions, in the pathogenesis of various cardiovascular diseases.

## 2. General Consideration of the Role of AGEs in Cardiovascular Diseases

It is generally considered that AGEs lead to the development of cardiovascular dysfunction in various diseases by generating oxidative stress and inflammation, as well as the cross-linking of proteins [41,42,43,44,45]; however, the exact mechanisms for the induction of adverse effects of AGEs in diverse diseases are not fully understood. Furthermore, in view of the heterogenous nature of compounds in different AGE groups, it is not clear whether each compound exerts similar actions in cardiac and vascular myocytes. It should be pointed out that reactive oxygen species (ROS) have been observed to promote the production of AGEs by accelerating the oxidation of sugars and accumulation of α-dicarbonyl compounds in the heart [46]. ROS have also been reported to produce the accumulation of AGEs upon lipid peroxidation through the reaction of aldehydes (malondialdehyde and 4-hydroxy-2-nonenal) with amino groups of proteins and nucleic acids in age-related cardiovascular disease [47]. Since, in addition to AGE–RAGE interaction, the generation of excessive amounts of ROS is also known to occur through other sources in various cardiovascular diseases [48], caution should be taken while interpreting the involvement of AGEs as a prime source of oxidative stress in the development of cardiovascular dysfunction.

Several members of the AGE groups have been reported to induce cardiovascular dysfunction due to their ability to create cross-links with different proteins and exhibit fluorescence upon cross-linking [49]. For example, cross-linking AGEs such as pentosidine and glyoxal-lysine dimers exhibit fluorescent properties, making them useful as biomarkers for the occurrence of oxidative stress and cellular damage. Some non-fluorescent cross-linking AGEs, such as glucosepane and imidazolium dilysine, which are abundant in the extracellular matrix, have also been shown to impair vascular compliance and contribute to tissue stiffness [41]. On the other hand, some non-cross-linking AGEs, such as N-carboxymethyl-lysine and pyralline, serve as surrogate markers for AGE accumulation due to their stability and detectability [50]. Recent studies have indicated the role of highly cross-linked AGEs, which are less abundant but more structurally disruptive, in impairing protein function, altering extracellular matrix composition, and contributing to vascular stiffening, cardiac fibrosis, and endothelial dysfunction [51,52,53,54,55]. It has also been emphasized that AGEs interact with RAGEs to induce the impairment of cardiac and vascular compliance in addition to promoting endothelial dysfunction, atherosclerosis, and myocardial remodeling [56,57,58]. By disrupting extracellular matrix proteins such as collagen and elastin, AGEs have been shown to contribute to the induction of arterial stiffness and cardiac fibrosis. There is also evidence to suggest that AGE deposition in cardiovascular tissues correlates with disease progression in conditions like diabetes mellitus, hypertension, and heart failure [59,60]. It is thus evident that AGEs are key mediators of cardiovascular diseases which originate from endogenous metabolic processes, inflammatory systems, oxidative stress, and exogenous dietary sources.

## 3. AGE–RAGE Interaction and Signaling Pathways in Cardiovascular Dysfunction

It should be mentioned that RAGEs are multiligand receptors, which are expressed in various cell types within the cardiovascular system [56]. RAGEs have a distinct structure, composed of a ligand-binding domain, a single transmembrane region, and an intracellular domain, which is involved in signal transduction. The extracellular domain of RAGEs binds to AGEs, as well as other ligands, including amyloid-beta, high-mobility group box 1 (HMGB1), and S100 proteins [61]. The expression of RAGE is tightly regulated at both the transcriptional and post-transcriptional levels. Under normal physiological conditions, RAGEs are expressed at low levels, but their expression increases in response to hyperglycemia, oxidative stress, and inflammation, which are characteristic features of the development of cardiovascular disease [62]. The binding of AGEs to RAGEs activates a cascade of intracellular signaling pathways that play a crucial role in the pathogenesis of cardiovascular diseases. Upon AGE binding, RAGEs undergo conformational changes that facilitate the recruitment of adapter proteins such as MyD88 and TRAF-6. These proteins activate the following downstream signaling pathways, namely nuclear factor-kappa B (NF-κB), mitogen-activated protein kinases (MAPKs), Janus kinase/signal transducers, and activators of transcription (JAK/STAT) pathways [63,64,65,66]:

(i) *NF-κB Pathway:* The activation of RAGEs triggers the NF-κB signaling pathway, a critical mediator of inflammation. NF-κB transcription factors, such as p65 and p50, translocate to the nucleus and promote the expression of pro-inflammatory cytokines, adhesion molecules, and other inflammatory mediators. Chronic activation of NF-κB by AGEs leads to endothelial dysfunction, increased vascular permeability, and recruitment of inflammatory cells to sites of injury; these alterations contribute to the development of atherosclerosis [67,68,69].
(ii) *MAPK Pathway:* RAGE activation also triggers the MAPK pathway, including p38 MAPK, extracellular signal-regulated kinase (ERK), and c-Jun N-terminal kinase (JNK). These kinases regulate gene expression and cellular responses such as cell proliferation, apoptosis, and inflammation. In the context of cardiovascular diseases, MAPK activation promotes the phenotypic switch of vascular smooth muscle cells, a key event in the development of atherosclerotic plaque [70,71,72].
(iii) *JAK/STAT Pathway:* The JAK/STAT signaling pathway is also implicated in RAGE-mediated effects. Activation of JAK1 and JAK2 leads to the phosphorylation and activation of STAT3, which subsequently modulates the expression of genes involved in inflammation and cell survival. This pathway contributes to the endothelial dysfunction observed in cardiovascular diseases, promoting a pro-inflammatory and pro-thrombotic state [73,74].

The interaction of AGEs with RAGEs initiates a feedback loop (the AGE–RAGE axis) which amplifies oxidative stress and inflammation in the development of cardiovascular diseases. Upon RAGE activation, the production of reactive oxygen species (ROS) is increased, further contributing to oxidative damage [75,76]. The generation of ROS, in turn, activates various transcription factors such as NF-κB, which induces the expression of pro-inflammatory cytokines and adhesion molecules for perpetuating the cycle of inflammation [32,58]. Moreover, AGE accumulation leads to the modification of extracellular matrix proteins, promoting tissue fibrosis and vascular stiffness, which are hallmarks of various cardiovascular diseases [77]. The AGE–RAGE axis is also involved in the formation of plaques in atherosclerosis. AGEs promote the oxidation of low-density lipoproteins (LDL), which accelerate the formation of foam cells and the progression of atherosclerotic lesions [78]. The binding of AGEs to RAGEs on macrophages and endothelial cells increases the expression of matrix metalloproteinases (MMPs), which degrade the extracellular matrix, destabilize atherosclerotic plaque, and increase the risk of plaque rupture as well as thrombosis [79].

Several other studies have also examined the role of the AGE–RAGE axis in activating a cascade of intracellular signaling events for the generation of oxidative stress and inflammation in endothelial cells, vascular smooth muscle cells, and cardiomyocytes [80,81,82,83,84]. The formation of ROS due to the interaction of AGEs with RAGEs has been reported to activate multiple pathways including NF-kβ, MAPK, and JAK-STAT; this induces the expression of inflammatory cytokines such as tumor necrosis factor-alpha (TNF-α) and intraleukin-6 (IL-6). These cytokines not only promote inflammation but also contribute to the development of endothelial dysfunction, impaired nitric oxide (NO) production, and increased vascular permeability. As a result, vascular smooth muscle cell proliferation and migration are stimulated, leading to the progression of atherosclerosis [80,81,82]. The increased production of ROS due to the activation of the AGE–RAGE axis contributes to lipid peroxidation, as well as the formation of oxidized LDL (oxLDL) and endothelial dysfunction, leading to the occurrence of myocardial cell injury, impaired cardiac contractility, and progression to heart failure [83]. Furthermore, AGE–RAGE interaction has been shown to modulate vascular aging, promote cellular senescence, and impair endothelial repair mechanisms [84]. Such observations have provided insight into the potential use of various interventions such as AGE inhibitors, RAGE antagonists, and antioxidants in mitigating the detrimental effects of the AGE–RAGE-associated signal transduction pathway in cardiovascular disease [80,81,82,83,84].

While within RAGEs, membrane-bound receptors for AGEs are involved in inducing adverse actions, other AGE-binding proteins have been identified which play a role in AGE clearance and in modulating AGE-induced cell damage [85,86,87,88,89]. These proteins in the circulatory system are either soluble RAGEs (sRAGEs), which are formed upon the proteolytic cleavage of membrane-bound RAGEs after activation of matrix metalloproteinase, or endogenous secretory RAGEs (esRAGEs), which are secreted from the cells and are spliced variants of RAGEs [40]. It should be mentioned that sRAGEs are a truncated form of membrane-bound RAGEs; they lacks the transmembrane domain, but retain the ligand-binding domain. These is released into the circulatory system and act as a decoy receptor upon binding with AGEs, preventing their interaction with membrane-bound RAGEs. As a result, sRAGEs can mitigate the pro-inflammatory and pro-oxidative effects of AGE accumulation. Elevated levels of sRAGEs are considered a protective mechanism in cardiovascular diseases, and their measurement may serve as a biomarker for assessing the risk of cardiovascular disease [85,86]. In addition, other AGE-binding proteins, such as esRAGEs or scavenger receptor A (SR-A), are primarily involved in the clearance of AGEs from the circulatory system [87,88,89]. They also play a role in modulating the inflammatory response to AGE accumulation. Other AGE receptors such as AGE-R1, AGE-R2, and AGE-R3 have also been reported as being present in cardiovascular tissues, and it has been claimed that these play a role in the endocytosis of AGEs for their detoxification [11]. However, sufficient information concerning these receptors is not available to make any conclusion regarding their exact function. Nonetheless, the involvement of membrane-bound RAGEs, sRAGEs, esRAGEs, and other AGE receptors in cardiovascular diseases highlights the complex network of AGE receptors in the pathogenesis as well as the treatment of cardiac dysfunction in cardiovascular ailments [58,87,88,89].

## 4. The Role of AGEs in Ischemic Heart Disease

Diabetes mellitus is a well-established risk factor for the accelerated progression of atherosclerosis, contributing to increased incidence, severity, and poor outcomes of ischemic heart disease, including myocardial infarction and stroke [90]. The pathogenic involvement of AGEs in the development of ischemic heart disease has been extensively demonstrated, particularly through their interaction with RAGEs [91]. A murine model of atherosclerosis has revealed an increased expression of RAGEs and their ligands in atherosclerotic plaque, particularly within macrophages, smooth muscle cells, and endothelial cells [73]. AGEs and other RAGE ligands, such as S100A12, calgranulins, and high-mobility group box 1 (HMGB1), are upregulated under hyperglycemic and inflammatory conditions, amplifying vascular injury and plaque progression. In hyperglycemic states, elevated AGE accumulation and increased expression of S100A12 have been observed within plaque, exacerbating the inflammatory milieu and promoting plaque instability [92]. Mechanistically, the activation of RAGEs by AGEs enhances inflammatory signaling pathways that are pivotal to atherogenesis. The binding of AGEs to RAGEs results in the downstream activation of NF-κB, leading to the upregulation of pro-inflammatory cytokines, adhesion molecules such as ICAM-1 and VCAM-1, and matrix metalloproteinases (MMPs), which contribute to endothelial dysfunction, monocyte recruitment, and plaque vulnerability [93].

In a murine model of diabetes-accelerated atherosclerosis, apolipoprotein E (apoE)-deficient mice treated with streptozotocin (type 1 diabetes) or bred into the db/db background (type 2 insulin-resistant diabetes), the administration of sRAGEs has been shown to significantly reduce the progression of atherosclerosis [94]. Importantly, the beneficial effects of sRAGEs occur independently of glycemic control, cholesterol levels, or triglyceride concentrations. The anti-atherosclerotic effects of sRAGEs are largely mediated by their ability to suppress vascular inflammation and oxidative stress, both of which are amplified in diabetes. Even in non-diabetic apoE-deficient mice, sRAGE administration reduces plaque burden, albeit to a lesser extent than in diabetic models, underscoring the role of AGE–RAGE signaling in both diabetes-accelerated and basal atherosclerosis [24]. Further insight into the role of RAGEs was gained through genetic deletion experiments. In apoE-deficient or LDL-receptor-deficient mice, RAGE deletion significantly reduced the extent of atherosclerosis and vascular inflammation compared to wild-type controls, irrespective of diabetic status [33]. While RAGE deletion did not alter systemic glucose homeostasis or lipid levels, it effectively attenuated diabetes-associated vascular stress, particularly through the inhibition of the Rho-associated coiled-coil-containing protein kinase 1 (ROCK1) pathway. ROCK1 activation by transforming growth factor-β2 (TGF-β2) signaling promotes vascular remodeling, fibrosis, and inflammatory responses, all of which are mitigated in RAGE-deficient mice [95]. These findings highlight the critical role of RAGEs in mediating vascular inflammation in diabetic and non-diabetic atherosclerosis.

It is pointed out that AGE accumulation in ischemic myocardium further exacerbates tissue injury by impairing extracellular matrix integrity, increasing myocardial stiffness, and promoting fibrosis [79]. Cross-linked AGEs, such as glucosepane, form irreversible bonds within collagen fibers, reducing myocardial compliance and contributing to diastolic dysfunction [5]. Additionally, AGE-induced endothelial dysfunction compromises microvascular reperfusion and further aggravates ischemic damage. Notably, the therapeutic inhibition of AGE formation using pharmacological agents such as aminoguanidine has shown promise in reducing myocardial damage and improving cardiac outcomes in experimental models of I/R injury [96]. The impact of AGE–RAGE signaling also extends to the exacerbation of ischemia-reperfusion (I/R) injury. It is noteworthy that I/R injury in the heart occurs when blood flow is restored at delayed stages of ischemia, leading to a burst of oxidative stress, inflammation, and cell death, as well as impaired recovery of cardiac function [97]. Experimental studies investigating the role of RAGEs in I/R injury employed two distinct approaches: the pharmacological blockade of AGEs using sRAGE, and the genetic deletion of RAGEs [74,98,99]. Both approaches demonstrated significant cardioprotective effects, including reduced infarct size, improved myocardial function, and diminished inflammatory cell infiltration. Mechanistically, RAGE activation during I/R injury amplifies ROS production and NF-κB activation, which perpetuates a cycle of oxidative stress, mitochondrial dysfunction, and inflammatory damage [100]. These observations are consistent with the view that the AGE–RAGE axis is not only involved in accelerating the I/R injury, but can also be a critical target for developing treatment for ischemic heart disease.

## 5. The Role of AGEs in Vascular Dysfunction

Vascular dysfunction induced by AGEs has emerged as a pivotal mechanism underlying the development and progression of hypertension and other cardiovascular diseases, particularly in aging and diabetes. One critical consequence of AGE formation is the cross-linking of extracellular matrix proteins, such as collagen, elastin, and laminin [48]. AGE-induced collagen cross-linking reduces the elasticity of vascular walls and myocardial tissues, increasing arterial stiffness and contributing to elevated systolic blood pressure and diastolic dysfunction [101]. These changes are frequently observed in aging populations and individuals with chronic diabetes. AGE-modified elastin and laminin impair endothelial cell adhesion, migration, and proliferation, exacerbating vascular remodeling and dysfunction [102]. Additionally, AGEs inhibit NO bioavailability by reducing eNOS activity and enhancing the scavenging of NO by ROS. This action diminishes vasodilation, promotes endothelial dysfunction and contributes to hypertension [103]. At the intracellular level, AGEs disrupt Ca^2+^ homeostasis in vascular smooth muscle cells and cardiomyocytes, leading to impaired vascular contractility and cardiac relaxation [15,27]. AGE-induced cross-linking of the SERCA inhibits its ability to accumulate Ca^2+^ and impairs diastolic relaxation [104]. Simultaneously, AGE-mediated modification of the RyR leads to abnormal calcium release and disruption of cardiomyocyte contraction. Furthermore, dysregulation of Ca^2+^ signaling enhances vascular smooth muscle proliferation and calcification processes which are central to arterial stiffness [105].

Several proteins in the circulatory system are also subject to AGE modifications, leading to functional impairment and promoting vascular disease. For instance, glycation of apolipoprotein B100 alters LDL structure, reducing its recognition by LDL receptors and promoting LDL accumulation in the bloodstream [106]. This facilitates the uptake of LDL by macrophages, leading to foam cell formation, lipid-laden plaques, and atherosclerosis. Similarly, glycated fibrinogen becomes resistant to proteolysis, impairing the fibrinolytic system and contributing to prothrombotic states. Such alterations significantly increase the risk of thrombotic events, including myocardial infarction and stroke, in diabetic and elderly populations [107]. Pharmacological agents such as ALT-711 (alagebrium), which break pre-formed AGE cross-links, have been demonstrated to improve arterial stiffness and endothelial function in previous experimental studies [108,109]. Additionally, interventions such as N-acetylcysteine [110] and metformin [111] have also been reported to reduce oxidative stress and suppress AGE–RAGE signaling, providing secondary benefits in vascular protection.

## 6. The Role of AGEs in Pathological Cardiac Hypertrophy and Heart Failure

Elevated AGE accumulation has been implicated in the maladaptive cardiac hypertrophy which invariably precedes the development of heart failure. In fact, the treatment of H9c2 cells with glyceraldehyde-derived AGEs was observed to induce cellular hypertrophy via the MEK/ERK pathway [112]. Increased ROS production was found to enhance oxidative damage to cellular proteins, lipids, and DNA which further accelerated cardiomyocyte death and fibrotic remodeling. AGE-induced activation of TGF-β/SMAD signaling also promoted fibroblast-to-myofibroblast transition, extracellular matrix deposition, and myocardial stiffness, which contributed to diastolic dysfunction [113]. Additionally, AGEs were shown to impair mitochondrial function upon reducing adenosine triphosphate production, exacerbate cardiomyocyte energy deficits and produce contractile dysfunction [114]. AGEs were also reported to play a critical role in the pathogenesis of cardiac hypertrophy by inducing extracellular matrix cross-linking [115], disrupting intracellular calcium homeostasis [116], and triggering chronic inflammation and oxidative stress through AGE–RAGE signaling [117]. Thus, targeting these pathways offers significant therapeutic potential for preventing and managing the development of cardiac hypertrophy or heart failure at early stages. In this regard, some pharmacological inhibitors of AGE formation, such as myriocin [114], and compounds targeting AGE–RAGE signaling, such as sRAGE and RAGE antagonists, have shown promise in reducing AGE-induced maladaptive cardiac hypertrophy and fibrosis.

AGE accumulation has been shown to be associated with the pathophysiology of heart failure, a complex clinical syndrome characterized by impaired cardiac function, often as a result of different comorbidities such as diabetes, hypertension, and coronary artery disease [118,119]. The role of AGEs in heart failure extends beyond mere glycation, involving intricate molecular and cellular mechanisms that drive adverse myocardial remodeling, inflammation, and cardiac dysfunction [120]. It has been shown that AGEs alter the composition and mechanical properties of the extracellular matrix, primarily through the cross-linking of collagen and elastin [121]. This cross-linking leads to increased myocardial stiffness, reduced compliance, and impaired myocardial relaxation [115]. These alterations are particularly pronounced in heart failure with preserved ejection fraction (HFpEF), where the inability of the left ventricle to fill effectively during diastole results from both AGE-induced cardiac remodeling and impaired cardiomyocyte relaxation [122]. Glycation of SERCA and RyR 2 proteins impair Ca^2+^-handling in cardiomyocytes [15,123] and contribute to both systolic and diastolic dysfunctions, characteristic features of heart failure with reduced ejection fraction (HFrEF) and HFpEF, respectively [123].

Patients with chronic diabetes were observed to exhibit increased myocardial AGE deposition, which correlated well with ventricular hypertrophy, fibrosis, and impaired cardiac function [124]. AGE–RAGE signaling exacerbates these pathological processes, underscoring AGEs as significant contributors to diabetic heart failure [125]. Some studies have also indicated that circulating AGEs such as N-carboxymethyl-lysine and pentosidine are associated with adverse outcomes in diabetes and heart failure [126]. Pharmacological approaches that inhibit AGE formation, such as aminoguanidine and pyridoxamine, as well as compounds such as alagebrium that disrupt AGE cross-linking have shown potential in reducing myocardial stiffness and improving diastolic function in diabetic cardiomyopathy [127,128,129,130,131,132]. These studies revealed that AGEs play a multifactorial role in the pathogenesis of heart failure, impacting myocardial structure, inflammation, oxidative stress, and calcium homeostasis. Understanding the precise molecular mechanisms by which AGEs influence cardiac dysfunction progression is essential for identifying targeted therapies that can disrupt this pathological process. Future research focusing on clinical trials and novel interventions targeting AGE pathways may pave the way for more effective treatments in this challenging cardiovascular condition.

**Figure 1:**
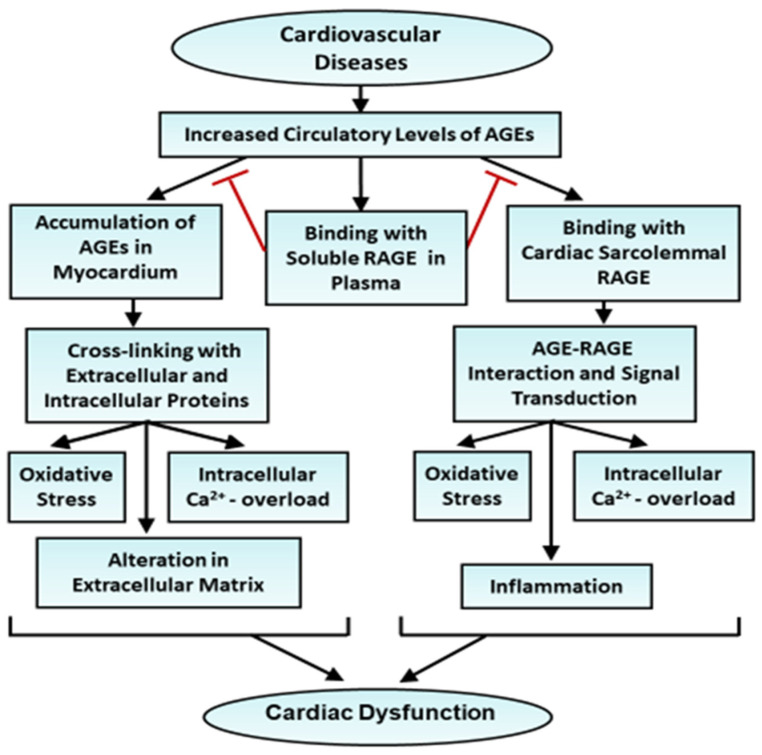
A schematic sketch of events in cardiovascular diseases indicating the effects of elevated levels of AGEs in the circulatory system for the induction of cardiac dysfunction AGEs (advanced glycation end-products); RAGEs, receptors for AGE.

In patients with HFpEF, the primary pathology involves impaired ventricular relaxation and increased stiffness, despite a normal ejection fraction. AGE accumulation in myocardial tissue exacerbates this dysfunction by promoting collagen cross-linking, leading to the stiffening of the heart muscle [105,133,134]. This stiffening results in impaired diastolic filling and increased left ventricular end-diastolic pressure, leading to symptoms of congestion and pulmonary edema. In addition to collagen cross-linking, AGEs contribute to impaired calcium handling and mitochondrial dysfunction, further aggravating the diastolic dysfunction characteristic of HFpEF. On the other hand, AGEs were observed to exacerbate systolic dysfunction in HFrEF through other mechanisms [135]. One key effect is the alteration of myocardial contractility due to the accumulation of AGEs in cardiac proteins, such as titin and myosin. AGE-induced cross-linking of these proteins impairs their function and leads to reduced contractile efficiency. Furthermore, AGEs increase oxidative stress and inflammation, which damages cellular structures, including the contractile apparatus and mitochondria, further contributing to cardiac dysfunction in patients with HFrEF [136,137]. AGEs have also been reported to induce cross-linking in extracellular matrix proteins, leading to increased myocardial stiffness and impaired relaxation [138]. Additionally, AGEs are known to interact with RAGE and activate signaling pathways for inducing cardiac remodeling and dysfunction in HFrEF [64]. Thus, it is likely that AGEs may contribute to the development of heart failure through cross-linking with different cardiac proteins as well as the activation of RAGE-mediated pathways. A simplified schematic sketch of events involving elevated levels of AGEs in the development of heart failure in various cardiovascular diseases is shown in Figure 1.

## 7. Pathophysiological and Therapeutic Implications of the AGE–RAGE Axis

Prasad and his associates have carried out extensive studies in defining the role of AGE–RAGE interaction and its implications for developing strategies for the treatment of several cardiovascular diseases [4,35,36,37,38,39,40]. These investigators have observed that the plasma levels of AGEs and the expression of RAGEs in vascular smooth muscle cells and cardiomyocytes were increased in patients with hypertension [139,140], aortic aneurysm [141,142], coronary artery disease [143,144], and acute myocardial infarction [145,146]. Furthermore, the plasma levels of sRAGEs were decreased in cardiovascular diseases, except for chronic diabetes and renal disease [4,22,36,37,38,39,40,141]. However, the ratio of plasma AGE/sRAGEs was increased in all cardiovascular diseases. The activation of AGE–RAGE or AGE–RAGE stress in almost all cardiovascular diseases was associated with cross-linking with collagen, the generation of oxidative stress, reduction of NO, and increased expression of endothelin-1, TGF-β, inflammatory cytokines, and cell adhesion molecules, as well as the activation of sympathetic activity and NF-kβ [139,140,141]. Several drugs such as aminoguanidine, angiotensin-converting enzyme inhibitors, angiotensin-II receptor blockers, different statins, and metformin have been reported to reduce the formation of AGEs and used for the treatment of several cardiovascular diseases [35]. Randomized controlled trials involving mobile health app and telemedicine-based chronic disease management interventions have shown promise in improving health outcomes in hyperglycemia patients [147,148]. Since conventional treatments have limited glycemic control and often show various side effects, some investigators have recommended the use of alternative therapies such as personalized diets and herbal medicine in AGE–RAGE-related complications [149,150]. In addition, the administration of sRAGEs or agents such as rosiglitazone and Vitamin D, which increase the levels of plasma sRAGEs, have been used for the treatment of various cardiovascular diseases [35,151]. Nonetheless, these findings indicate that the AGE–RAGE axis is involved in several cardiovascular diseases; however, further research is needed to understand the cause–effect relationship of changes in the AGE–RAGE axis, as well as the pathogenesis of each cardiovascular disease.

## 8. Conclusions and Perspectives

AGEs have emerged as critical mediators in the pathogenesis of various cardiovascular diseases. The detrimental effects of AGEs are mediated through both structural modifications of extracellular and intracellular proteins by cross-linking as well as the activation of membrane-bound RAGEs, which triggers pro-inflammatory and pro-oxidative signaling pathways. In ischemic heart disease, AGEs exacerbate plaque instability and I/R injury, while in heart failure, they contribute to both preserved and reduced ejection fraction phenotypes by promoting myocardial stiffness, fibrosis, and impaired contractility. The role of AGEs in diabetic cardiomyopathy is particularly pronounced, where chronic hyperglycemia accelerates AGE accumulation, leading to severe cardiac remodeling and dysfunction. It should also be mentioned that various pharmacological agents such as aminoguanidine and alagebrium, as well as dietary interventions aimed at reducing AGE intake, have been shown to exert beneficial effects in some animal studies. However, extensive clinical studies in humans on AGE-related cardiovascular diseases need to be carried out in order to make any meaningful conclusion. Additionally, antioxidants and RAGE inhibitors offer avenues for reducing oxidative stress and inflammation, thereby preserving myocardial and vascular function. Understanding the molecular mechanisms underlying AGE-mediated damage provides a foundation for developing targeted therapies to combat cardiovascular diseases. Future research should focus on translating these findings into clinical practice, with an emphasis on novel approaches to reduce the burden of AGEs and improve cardiovascular outcomes in both diabetic and non-diabetic populations. By addressing the AGE–RAGE axis, we can pave the way for more effective prevention and treatment strategies, ultimately reducing the global burden of cardiovascular diseases.

From the information outlined in this article, it is evident that although the levels of AGEs are elevated in the circulatory systems of patients with diverse cardiovascular diseases, it is difficult to establish the exact role of any specific AGE compound in the pathogenesis of any particular cardiovascular ailment. This difficulty stems from the fact that the pathophysiology of cardiovascular diseases is of a complex nature, as several other toxic factors in addition to AGEs are known to be involved in the genesis of cardiovascular abnormalities. Furthermore, the mechanisms leading to the development of any particular cardiovascular disease are shown to differ. It is also not clear which AGE compound out of more than 20 AGEs is most effective in eliciting any particular cardiovascular disease. Thus, it would be prudent to examine the time-course of changes in the levels of AGEs in various cardiovascular diseases by employing improved specific methods for their determination. Efforts should also be made to develop specific AGE antagonists, and their effectiveness should be examined in both experimental and clinical studies. In view of the properties of sRAGEs and esRAGEs in antagonizing AGE–RAGE interactions in cardiac and vascular myocytes, efforts should be made to develop agents for promoting the expression of both of these soluble RAGE receptors. Likewise, extensive work should be carried out to establish the nature of AGE-R1, AGE-R2, and AGE-R3 for their beneficial effects in various cardiovascular diseases in addition to developing interventions for promoting their expression. It is hoped that such suggestions will be helpful in establishing the exact role of AGEs in pathogenesis, as well as in improving the treatment of cardiovascular diseases.

## Figures and Tables

**Table 1:** The classification, composition, and protein targets of some of the advanced glycation end-products (AGEs).

AGE Groups	Composition	Targets
**A. Fluorescent, cross-linked AGEs**		a. Targets for cross-linked AGEs in groups A and B:
1 Pentosidine:	Arginine and lysine residue cross-linked with ribose	(i) Cross-linking with extracellular matrix proteins: collagen, vitronectin and lamin.
2 Pentodilysine:	Lysine and lysine residues cross-linked with ribose	(ii) Cross-linking with cardiomyocyte and vascular proteins: collagen, sarcoplasmic reticulum Ca^2+^-ATPase and ryanodine receptors.
**B. Non-fluorescent, cross-linked AGEs**		
1 GOLD:	Glyoxal-lysine dimer	
2 MOLD:	Methylglycoxal-lysine dimer	
**C. Non-fluorescent, non-cross-linked AGEs**		b. Targets for non-cross-linked AGEs in groups C and D:
1 CEL:	Carboxyethyl-lysine	
2 CML:	Condensation of glucose with lysine of amino group	(i) Binding with RAGEs in macrophages, fibroblasts, endothelial cells, and Cardiomyocytes.
3 Pyrraline:	Reaction between glucose and lysine residues	(ii) Binding with sRAGEs and esRAGEs as well as AGE receptors such as AGE-R1, AGE-R2, and AGE-R3.
**D. Fluorescent, non-cross-linked AGEs**		
1 Argpyrimidine:	Formed from arginine and methylglycoxal	

More than 20 AGEs have been identified in human blood and tissues [7,8,9,10,11]; we have selected 8 AGEs as examples for different classification groups. The chemical structures of different AGEs are shown by previous investigators [7,8]. Furthermore, the identification of target sites for different AGEs is based on the information regarding the interaction of AGEs for cross-linking with different proteins as well as binding with different AGE receptors for the induction of cardiovascular effects [7,8,9,10,11].

## Data Availability

No new data were created or analyzed in this study.
